# How Can We Achieve Healthy Aging?

**DOI:** 10.3390/ijerph14121583

**Published:** 2017-12-15

**Authors:** Jean Woo

**Affiliations:** Department of Medicine and Therapeutics and CUHK Jockey Club Institute of Aging, The Chinese University of Hong Kong, Hong Kong, China; jeanwoowong@cuhk.edu.hk

Population aging affects all countries, and all income groups. To date, epidemiologists and public health professionals have contributed to this discourse by examining the impact of non-communicable diseases, while gerontologists and geriatricians have been engaged in developing the study of frailty, which may represent a better public health indicator of aging well, and may be regarded as a measure of intrinsic capacity in older people, using a more negative descriptor. There has been little cross-talk between the two disciplines, resulting in a situation that does not allow society to meet the needs of older people in a responsive way. The World Health Organization provided an impetus with the publication of the first World Report on Aging and Health in 2015, which emphasizes functional capacity as a main goal rather than mortality [1]. It further proposes that functional capacity may be achieved by a combination of promoting (or retarding the decline) of intrinsic capacity. The latter is a product of personal factors such as socioeconomic status, education, and lifestyle, together with physical and social environmental factors. Thus, our response to population aging should incorporate these principles, at social and health policy levels, as well as further downstream to units that provide direct care. The articles in this special issue contribute to different facets in this discourse.

The World Health Organization is at the same time promoting the Age-Friendly City movement, in which over 300 countries have participated to date [2]. This concept is a major contributor to promoting functional capacity. This area is explored in the papers by Tiraphat S. et al., Portegijs E. et al., Aboderin I. et al., Wong M. et al., Yu R. et al., and Ho H.C. et al. [3,4,5,6,7,8]. The importance of social environment is discussed by Lotvonen S. et al. as well as Wong A. et al. [9,10]. Analysis of factors contributing to frailty in two Chinese populations with different profiles and health and social care systems provide more in-depth insight regarding strategies for the prevention of frailty or for maintaining intrinsic capacity [11]. How technology may be used is explored by Santa-Mancilla et al. [12]. Yamada et al. [13] addresses a more downstream issue relating to interventions for healthy life expectancy. With respect to policy and the organization of health and social care systems, an ideal design to meet current needs is discussed [14]; meanwhile, the Singapore government has already formulated policy to tackle frailty [15]. Finally, an intriguing exploration of blue zones in China provides further stimulus for carrying out research on the role of nutritional and other environmental factors in healthy aging [16].

## References

[1] Beard J.R., Officer A., de Carvalho I.A., Sadana R., Pot A.M., Michel J.P., Lloyd-Sherlock P., Epping-Jordan J.E., Peeters G.M.E.E., Mahanani W.R. (2016). The World report on ageing and health: A policy framework for healthy ageing. Lancet.

[2] WHO Global Network for Age-Friendly Cities and Communities. http://www.who.int/ageing/projects/age_friendly_cities_network.

[3] Tiraphat S., Peltzer K., Thamma-Aphiphol K., Suthisukon K. (2017). The Role of Age-Friendly Environments on Quality of Life among Thai Older Adults. Int. J. Environ. Res. Public Health.

[4] Portegijs E., Keskinen K.E., Tsai L.T., Rantanen T., Rantakokko M. (2017). Physical Limitations, Walkability, Perceived Environmental Facilitators and Physical Activity of Older Adults in Finland. Int. J. Environ. Res. Public Health.

[5] Aboderin I., Kano M., Owii H.A. (2017). Toward “Age-Friendly Slums”? Health Challenges of Older Slum Dwellers in Nairobi and the Applicability of the Age-Friendly City Approach. Int. J. Environ. Res. Public Health.

[6] Wong M., Yu R., Woo J. (2017). Effects of Perceived Neighbourhood Environments on Self-Rated Health among Community-Dwelling Older Chinese. Int. J. Environ. Res. Public Health.

[7] Yu R., Cheung O., Lau K., Woo J. (2017). Associations between Perceived Neighborhood Walkability and Walking Time, Wellbeing, and Loneliness in Community-Dwelling Older Chinese People in Hong Kong. Int. J. Environ. Res. Public Health.

[8] Ho H.C., Lau K.K.L., Yu R., Wang D., Woo J., Kwok T.C.Y., Ng E. (2017). Spatial Variability of Geriatric Depression Risk in a High-Density City: A Data-Driven Socio-Environmental Vulnerability Mapping Approach. Int. J. Environ. Res. Public Health.

[9] Lotvonen S., Kyngas H., Koistinen P., Bloigu R., Elo S. (2017). Social Environment of Older People during the First Year in Senior Housing and Its Association with Physical Performance. Int. J. Environ. Res. Public Health.

[10] Wong A., Chau A.K.C., Fang Y., Woo J. (2017). Illuminating the Psychological Experience of Elderly Loneliness from a Societal Perspective: A Qualitative Study of Alienation between Older People and Society. Int. J. Environ. Res. Public Health.

[11] Yu R., Wu W.C., Leung J., Hu S.C., Woo J. (2017). Frailty and Its Contributory Factors in Older Adults: A Comparison of Two Asian Regions (Hong Kong and Taiwan). Int. J. Environ. Res. Public Health.

[12] Santana-Mancilla P.C., Anido-Rifon L.E. (2017). The Technology Acceptance of a TV Platform for the Elderly Living Alone or in Public Nursing Homes. Int. J. Environ. Res. Public Health.

[13] Yamada M., Arai H. (2017). Self-Management Group Exercise Extends Healthy Life Expectancy in Frail Community-Dwelling Older Adults. Int. J. Environ. Res. Public Health.

[14] Woo J. (2017). Designing Fit for Purpose Health and Social Services for Ageing Populations. Int. J. Environ. Res. Public Health.

[15] Lim W.S., Wong W.F., Leong I., Choo P., Pang W.S. (2017). Forging a Frailty-Ready Healthcare System to Meet Population Ageing. Int. J. Environ. Res. Public Health.

[16] Huang Y., Jacquez G.M. (2017). Identification of a Blue Zone in a Typical Chinese Longevity Region. Int. J. Environ. Res. Public Health.

